# Towards personalized medicine for refractory/relapsed follicular lymphoma patients: The Lupiae‐Cantera study

**DOI:** 10.1002/hem3.70230

**Published:** 2025-10-08

**Authors:** Irene Dogliotti, Sanne Tonino, Luana Conte, Yana Stepanishyna, Filipa Moita, Sofia Brites Alves, Ana Jiménez‐Ubieto, Sonia Gonzalez de Villambrosia, Federica Cavallo, Raquel Del Campo Garcia, Natalia Zing, Marie José Kersten, Massimo Federico, The EHA LyG Cantera 2018

**Affiliations:** ^1^ Department of Biotechnology and Health Sciences, Division of Hematology University of Torino Torino Italy; ^2^ Department of Hematology Amsterdam UMC Location University of Amsterdam Amsterdam The Netherlands; ^3^ Department of Physics and Chemistry University of Palermo Palermo Italy; ^4^ Laboratory of Advanced Data Analysis for Medicine (ADAM) at DReAM University of Salento and ASL (Local Health Authority), “V. Fazzi” Hospital Lecce Italy; ^5^ Department of Hematology Jules Bordet Institute Brussels Belgium; ^6^ Instituto Português de Oncologia Francisco Gentil Lisbon Portugal; ^7^ Servicio de Hematología Hospital 12 de Octubre Madrid Spain; ^8^ Hospital Universitario Marqués de Valdecilla (IDIVAL) Santander Spain; ^9^ Hematology Department Hospital Son Llàtzer Palma de Mallorca Islas Baleares Spain; ^10^ Hematology Department Hospital Son Llàtzer, IdisBa Palma de Mallorca Islas Baleares Spain; ^11^ Department of Onco‐Hematology Hospital Beneficência Portuguesa de São Paulo São Paulo Brazil; ^12^ T‐Cell Brazil Project Brazil; ^13^ CHIMOMO Department University of Modena and Reggio Emilia Modena Italy

Follicular lymphoma (FL) is the second most common non‐Hodgkin lymphoma (NHL) in Western countries, accounting for about 10%–20% of all newly diagnosed NHLs and 70% of all indolent lymphomas.^1^, ^2^

The clinical course of FL is typically indolent and is characterized by a waxing and waning course. Most patients eventually need treatment, and responses to initial chemo‐immunotherapy (CIT) are usually impressive. Nevertheless, relapses occur, requiring additional therapeutic interventions that result in shorter remission duration and an increased risk of drug resistance.^3^ At present, progression‐free survival (PFS) after CIT in advanced stage FL ranges from 73% to 86% at 3 years,^4^, ^5^ and overall survival (OS) at 5 years varies between 68% and 90%, depending on the patient's age group.^6^, ^7^

Disease progression or relapse within 2 years from first‐line CIT (POD24_1) identifies a group of FL patients with significantly inferior outcomes (12% event‐free survival at 5 years)^6^, ^8^ and hence with an unmet medical need. Current knowledge is insufficient to identify patients with high‐risk disease upfront, nor can it guide initial treatment decisions. Second‐line treatments employed in patients with relapsed/refractory (R/R) FL, which differ greatly from country to country and even within single institutions, are guided by initial therapy, patient's age and fitness, and disease characteristics.^9^, ^10^, ^11^, ^12^

For the design of more uniform treatment guidelines, it is important to better understand which specific combinations of first‐ and second‐line treatments result in the most favorable outcome in specific FL patient populations.

Thanks to the extraordinary commitment of Dr. Steve Ansell, the Coach of the Cantera—2018 edition, and his fantastic training ability, it took just a few days for 20 individuals (the Cantera Players) to become one single, compact group: the Lupiae team (Figure 1).

**Figure 1:**
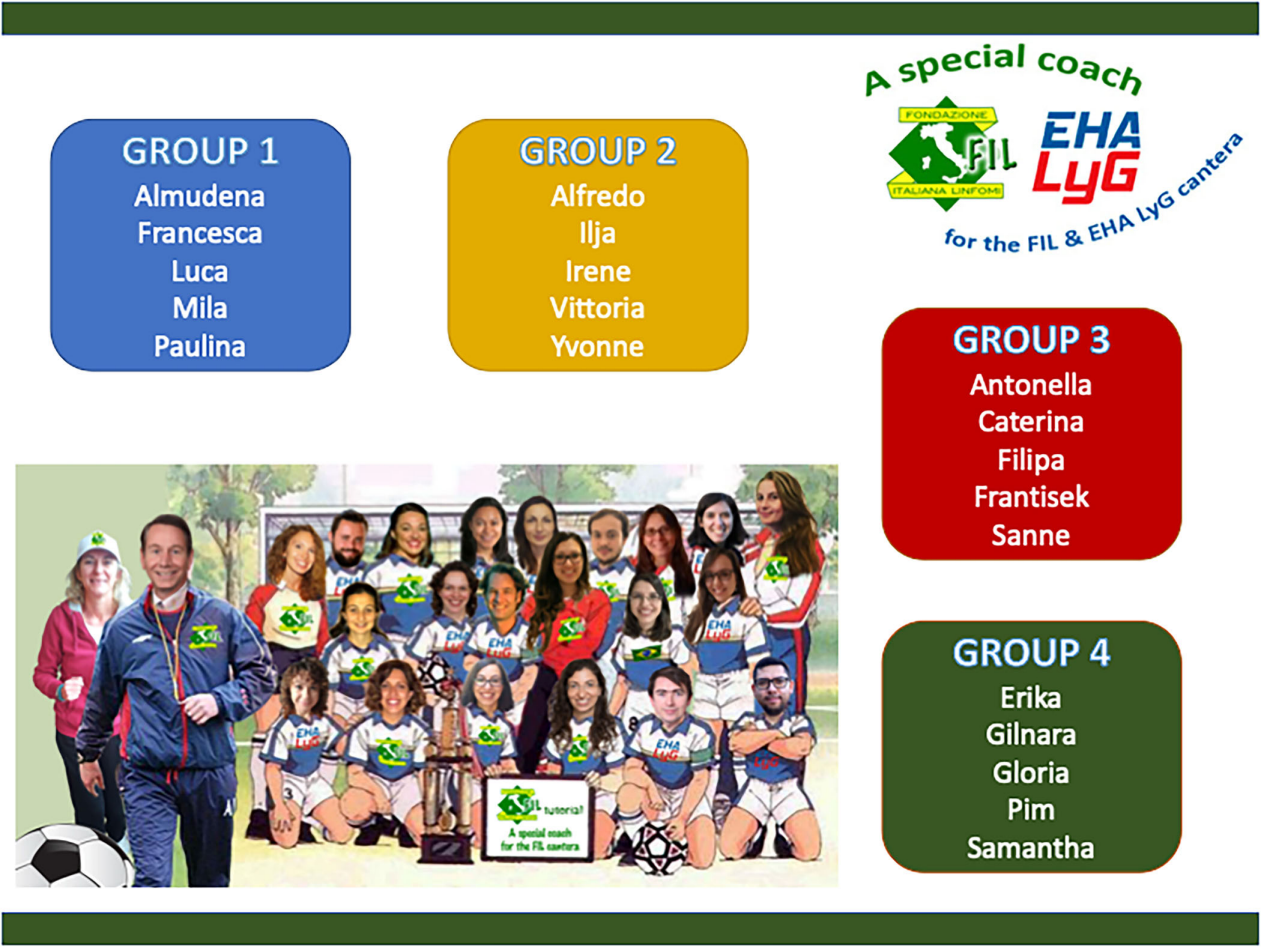
The 2018 EHA LyG Cantera team (coach, assistant coach, and players).

The magic blend of these young brains soon produced the Lupiae study, an observational study whose aim was to define the disease course of R/R FL after first‐line CIT, report current real‐life approaches in various countries, and provide a rationale for the identification of novel treatment strategies. The Cantera Headquarter and EHA LyG enthusiastically supported this project, and in March 2019, the LUPIAE registry (NCT04587388) opened enrollment.

Patients with a histologically confirmed initial diagnosis of Grades 1–3a FL who were refractory to first‐line CIT or who had relapsed or transformed to aggressive lymphoma were eligible and registered at the time of the first event (documented by biopsy, imaging, or clinical evaluation). Events were defined as (1) FL progression during induction or maintenance therapy; (2) FL relapse or progression after the achievement of at least partial remission (PR); and (3) transformation to aggressive B‐cell lymphoma. Patients are considered refractory in the absence of response (<PR) to first‐line CIT, while a relapse is defined as an initial response (at least PR) with subsequent disease reappearance or progression. In this real‐life study, since no genetic data were available, patients who reported having Grade 3b FL at the time of first event were considered as having transformed disease (tFL).

Patient registration was performed online in a key‐restricted database. Patients with the following criteria were excluded: (1) histological Grade 3b FL or transformed FL at initial diagnosis; (2) previous treatment with more than one systemic line of therapy. The principal endpoint was the rate of progression of disease at 24 months after second‐line treatment (POD24_2). As the study is noninterventional, a waiver was issued by the Ethics Committee of the coordinating center (Amsterdam UMC, the Netherlands) and by local Committees of participating centers per national regulations.

From March 2019 to November 2024, 160 consecutive R/R FL patients were registered at 22 academic and nonacademic sites in 10 different countries, 122 of whom were assessable for further analyses, including details on first‐ and second‐line therapy.

First‐line CIT consisted of rituximab/obinutuzumab combined with either cyclophosphamide, doxorubicin, vincristine and prednisone (R/GA‐CHOP), cyclophosphamide, vincristine, and prednisone (R‐CVP), or bendamustine (R/GA‐Benda), in 78 (63.9%), 15 (12.2%), and 16 (13.1%) patients, respectively; 76 (62.3%) of patients received rituximab maintenance after first‐line CIT. Complete responses, PR, and stable/progressive disease (SD/PD) were observed in 66%, 22%, and 12% of cases, respectively.

A first event was recorded as primary refractory disease in 30 patients (24.6%); among these, early transformation into aggressive lymphoma occurred in 6 patients (20% of the primary refractory patients). Of the 92 patients who initially responded (CR or PR), 12/92 (13%) relapsed as tFL, while 80 (87%) maintained indolent histology.

The median time between initial diagnosis and first event (TTFE) was 36 months (range 0–307); relapse/progression occurred within 24 months (POD24) in 33.6% (*n* = 41) of patients. TTFE was significantly impacted by response to first‐line treatment (59.0 vs. 29.0 vs. 6.5 months, respectively, for patients in CR, PR, or SD, P = 0.004). Among tFL patients, median TTFE was shorter compared to non‐transformed FL cases, although not reaching statistical significance (23.0 vs. 38.5 months, P = 0.06);

Of the 38 patients who progressed during initial CIT or maintenance, the first event occurred during CIT induction in 10 (26%); the majority (28 cases, 74%) occurred during anti‐CD20 maintenance.

At the time of the first event, 88% of enrolled patients underwent restaging with fluorodeoxyglucose (FDG)‐positron emission tomography (PET) scan (vs. 63% at initial diagnosis).

To date, data on second‐line treatment are available for 104 of the 122 patients. In contrast with the rather uniform first‐line approach, treatments at the time of first event were highly heterogeneous, with 15 different second‐line therapies (Figure 2).

**Figure 2:**
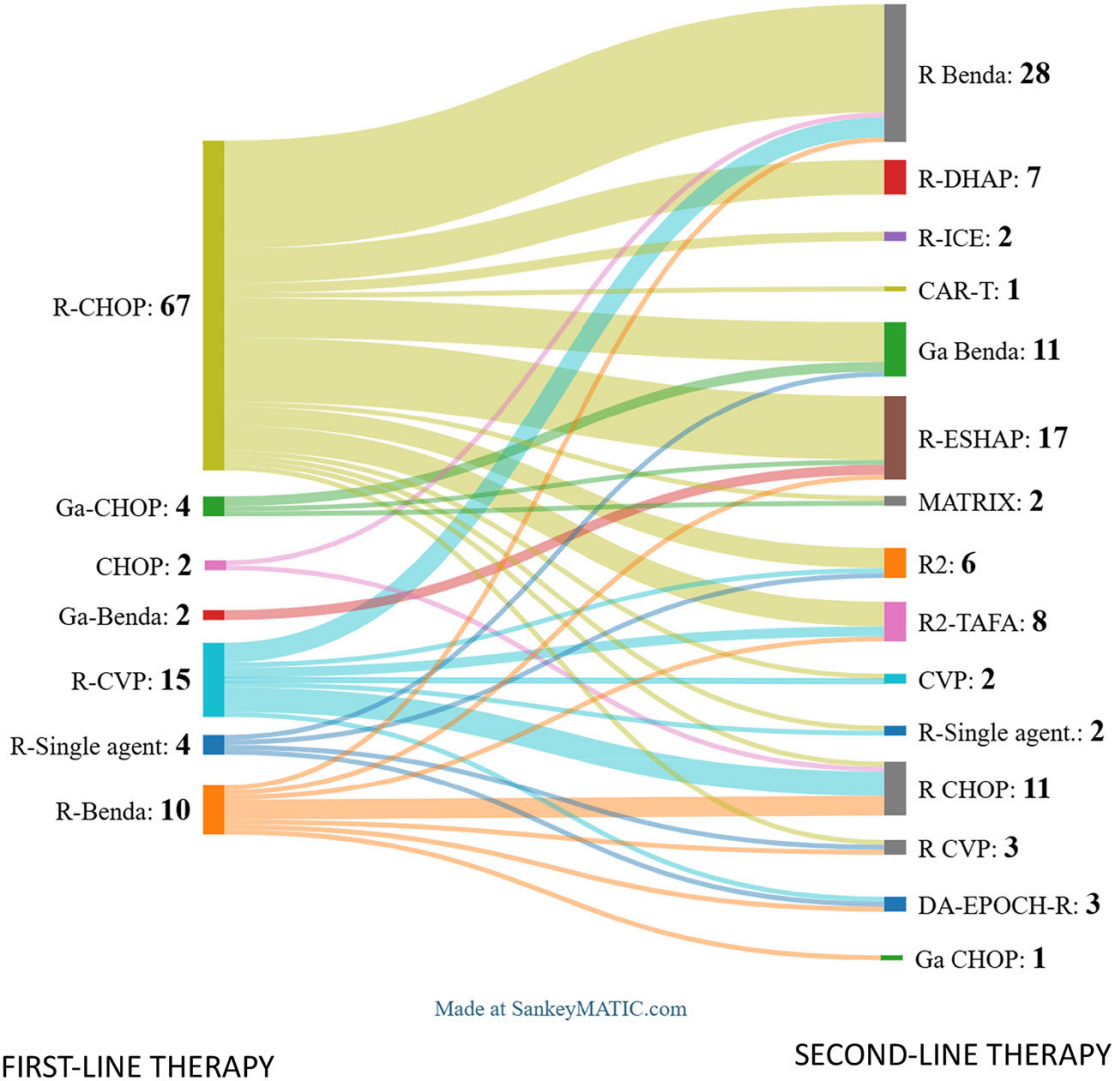
Sankey plot describing the trends in treatments employed in first and second line (included *n* = 104).

Although enrollment is ongoing, the preliminary results of the LUPIAE registry show that this prospective observational initiative provides valuable insights into the clinical course and current real‐life scenarios for patients with R/R FL in a wide variety of hospitals in 10 different countries. Most notably, these results clearly show the heterogeneity of available treatments and of guidelines, likely due to a lack of prospective randomized trials in this multifaceted disease.

A few other interesting preliminary findings stand out, such as the consistent use of the FDG‐PET scan, especially at relapse (87% of the whole cohort), and the variance in application of maintenance therapy (62% after first‐line, 25% after second‐line treatment).

In the future, more mature data from the Lupiae study on the efficacy of second‐line treatment regimens and PFS2 related to clinical variables will provide important information for the refinement of risk assessment and prognostication in FL as well as for the rational design of treatment algorithms, particularly for high‐risk patients.

## AUTHOR CONTRIBUTIONS

**Irene Dogliotti:** Conceptualization; investigation; writing—original draft; writing—review and editing; supervision; methodology. **Sanne Tonino:** Conceptualization; investigation; writing—original draft; writing—review and editing; supervision; methodology. **Luana Conte:** Writing—original draft; writing—review and editing; formal analysis; data curation. **Yana Stepanishyna:** Investigation; writing—original draft; formal analysis; writing—review and editing; supervision; methodology. **Filipa Moita:** Investigation; writing—original draft; visualization. **Sofia Brites Alves:** Investigation; writing—original draft. **Ana Jiménez‐Ubieto:** Investigation; writing—original draft. **Sonia Gonzales de Villambrosia:** Investigation; writing—original draft; visualization. **Federica Cavallo:** Investigation; supervision; writing—original draft; writing—review and editing; methodology. **Raquel Del Campo Garcia:** Investigation; visualization; writing—original draft. **Natalia Zing:** Investigation; visualization; writing—original draft. **Marie José Kersten:** Conceptualization; methodology; investigation; supervision; writing—original draft; writing—review and editing; formal analysis. **Massimo Federico:** Conceptualization; methodology; data curation; investigation; formal analysis; supervision; funding acquisition; resources; writing—original draft; writing—review and editing.

## CONFLICTS OF INTEREST STATEMENT

The authors declare no conflicts of interest.

## FUNDING

This research received no funding.

## Data Availability

The data that support the findings of this study are available on request from the corresponding author. The data are not publicly available due to privacy or ethical restrictions.

## References

[1] Swerdlow SH , Campo E , Pileri SA , et al. The 2016 revision of the World Health Organization classification of lymphoid neoplasms. Blood. 2016;127(20):2375‐2390. 10.1182/blood-2016-01-643569 26980727 PMC4874220

[2] Zelenetz AD , Gordon LI , Wierda WG , et al. Non‐Hodgkin's lymphomas, version 4.2014. J Natl Compr Cancer Netw. 2014;12(9):1282‐1303. 10.6004/jnccn.2014.0125 PMC4839265 25190696

[3] Tan D , Horning SJ , Hoppe RT , et al. Improvements in observed and relative survival in follicular grade 1‐2 lymphoma during 4 decades: the Stanford University experience. Blood. 2013;122(6):981‐987. 10.1182/blood-2013-03-491514 23777769 PMC3739040

[4] Luminari S , Manni M , Galimberti S , et al. Response‐adapted postinduction strategy in patients with advanced‐stage follicular lymphoma: the FOLL12 study. J Clin Oncol. 2022;40(7):729‐739. 10.1200/JCO.21.01234 34709880

[5] Marcus R , Davies A , Ando K , et al. Obinutuzumab for the first‐line treatment of follicular lymphoma. N Engl J Med. 2017;377(14):1331‐1344. 10.1056/NEJMoa1614598 28976863

[6] Day JR , Larson MC , Durani U , et al. Treatment patterns and outcomes in follicular lymphoma with POD24: an analysis from the LEO Consortium. Blood Adv. 2025;9(5):1013‐1023. 10.1182/bloodadvances.2024014053 39602301 PMC11909426

[7] Dinnessen MAW , van der Poel MWM , Tonino SH , et al. Stage‐specific trends in primary therapy and survival in follicular lymphoma: a nationwide population‐based analysis in the Netherlands, 1989–2016. Leukemia. 2021;35(6):1683‐1695. 10.1038/s41375-020-01048-6 33046819

[8] Casulo C , Byrtek M , Dawson KL , et al. Early relapse of follicular lymphoma after rituximab plus cyclophosphamide, doxorubicin, vincristine, and prednisone defines patients at high risk for death: an analysis from the National LymphoCare study. J Clin Oncol. 2015;33(23):2516‐2522. 10.1200/JCO.2014.59.7534 26124482 PMC4879714

[9] Dreyling M , Ghielmini M , Rule S , et al. Newly diagnosed and relapsed follicular lymphoma: ESMO Clinical Practice Guidelines for diagnosis, treatment and follow‐up. Ann Oncol. 2021;32(3):298‐308. 10.1016/j.annonc.2020.11.008 33249059

[10] Rossi G , Marcheselli L , Dondi A , et al. The use of anthracycline at first‐line compared to alkylating agents or nucleoside analogs improves the outcome of salvage treatments after relapse in follicular lymphoma The REFOLL study by the Fondazione Italiana Linfomi. Am J Hematol. 2015;90(1):56‐61. 10.1002/ajh.23872 25327841

[11] Casulo C , Barr PM . How I treat early‐relapsing follicular lymphoma. Blood. 2019;133(14):1540‐1547. 10.1182/blood-2018-08-822148 30700421

[12] Batlevi CL , Sha F , Alperovich A , et al. Follicular lymphoma in the modern era: survival, treatment outcomes, and identification of high‐risk subgroups. Blood Cancer J. 2020;10(7):74. 10.1038/s41408-020-00340-z 32678074 PMC7366724

